# Evidence of the impacts of metal mining and the effectiveness of mining mitigation measures on social–ecological systems in Arctic and boreal regions: a systematic map

**DOI:** 10.1186/s13750-022-00282-y

**Published:** 2022-09-08

**Authors:** Neal R. Haddaway, Adrienne Smith, Jessica J. Taylor, Christopher Andrews, Steven J. Cooke, Annika E. Nilsson, Pamela Lesser

**Affiliations:** 1 Stockholm Environment Institute, Linnégatan 87D, Stockholm, Sweden; 2 Leibniz-Centre for Agricultural Landscape Research (ZALF), Müncheberg, Germany; 3 Africa Centre for Evidence, University of Johannesburg, Johannesburg, South Africa; 4 Canadian Centre for Evidence-Based Conservation, Carleton University, 1125 Colonel by Drive, Ottawa, ON K1S 5B6 Canada; 5 Luleå University of Technology, 971 87 Luleå, Sweden; 6 Arctic Centre, University of Lapland, 96101 Rovaniemi, Finland

**Keywords:** Resource extraction, Extractive industries, Base metal mining, Mitigation effectiveness, Environmental impact, Social impact, Arctic biome

## Abstract

**Background:**

Mining can directly and indirectly affect social and environmental systems in a range of positive and negative ways, and may result in societal benefits, but may also cause conflicts, not least in relation to land use. Mining always affects the environment, whilst remediation and mitigation efforts may effectively ameliorate some negative environmental impacts. Social and environmental systems in Arctic and boreal regions are particularly sensitive to impacts from development for numerous reasons, not least of which are the reliance of Indigenous peoples on subsistence livelihoods and long recovery times of fragile ecosystems. With growing metal demand, mining in the Arctic is expected to increase, demanding a better understand its social and environmental impacts. We report here the results of a systematic mapping of research evidence of the impacts of metal mining in Arctic and boreal regions.

**Methods:**

We searched multiple bibliographic databases and organisational websites for relevant research using tested search strategies. We also collected evidence from stakeholders and rightsholders identified in the wider 3MK project (Mapping the impacts of Mining using Multiple Knowledges, https://osf.io/cvh3u). We screened articles at three stages (title, abstract, and full text) according to a predetermined set of inclusion criteria, with consistency checks between reviewers at each level. We extracted data relating to causal linkages between actions or impacts and measured outcomes, along with descriptive information about the articles and studies. We have produced an interactive database along with interactive visualisations, and identify knowledge gaps and clusters using heat maps.

**Review findings:**

Searches identified over 32,000 potentially relevant records, which resulted in a total of 585 articles being retained in the systematic map. This corresponded to 902 lines of data on impact or mitigation pathways. The evidence was relatively evenly spread across topics, but there was a bias towards research in Canada (35% of the evidence base). Research was focused on copper (23%), gold (18%), and zinc (16%) extraction as the top three minerals, and open pit mines were most commonly studied (33%). Research most commonly focused on operation stages, followed by abandonment and post-closure, with little evidence on early stages (prospecting, exploration, construction; 2%), expansion (0.2%), or decommissioning/closure (0.3%). Mitigation measures were not frequently studied (18% articles), with groundwater mitigation most frequently investigated (54% of mitigations), followed by soil quality (12%) and flora species groups (10%). Control-impact study designs were most common (68%) with reference sites as the most frequently used comparator (43%). Only 7 articles investigated social and environmental outcomes together. the most commonly reported system was biodiversity (39%), followed by water (34%), societies (20%), and soil/geology (6%), with air the least common (1%).

**Conclusions:**

The evidence found highlights a suite of potential knowledge gaps, namely: on early stages prior to operation; effectiveness of mitigation measures; stronger causal inference study designs; migration and demography; cumulative impacts; and impacts on local and Indigenous communities. We also tentatively suggest subtopics where the number of studies could allow systematic reviews: operation, post-closure, and abandonment stages; individual faunal species, surface water quality, water sediment quality; and, groundwater mitigation measure effectiveness.

**Supplementary Information:**

The online version contains supplementary material available at 10.1186/s13750-022-00282-y.

## Background

### On the impacts of mining

Mining activities, including prospecting, exploration, construction, operation, maintenance, expansion, abandonment, decommissioning and repurposing may affect social and environmental systems in a range of direct and indirect, positive and negative ways. Exploration, construction, operation, and maintenance of mines can change land-use substantially, and may negatively affect environments, for example through deforestation, erosion, contamination and alteration of soil profiles, contamination of local streams and wetlands, and an increase in noise level, dust and emissions [1–5]. Abandonment, decommissioning and repurposing of mines can also cause significant environmental impacts, for example soil and water contamination [e.g. 6–8. Additionally, infrastructure put in place to support mining activities (i.e. roads, ports, railway tracks, power lines) may affect migratory routes of animals and worsen habitat fragmentation [9, 10]. Such infrastructure, together with accompanying institutions, can indeed create new social-technological mega-systems [11–13].

Mining also affects people and societies. Negative effects include impacts on human health [e.g. 14 and living standards [15]. Mining is known to affect traditional practices of Indigenous peoples [16], and land-use conflicts are also often present, as are other societal impacts including those related to public health and human wellbeing [e.g. 17, 18–20. In terms of positive impacts, mining is typically a source of local employment and may contribute to economies locally and regionally [e.g. 21, 22. Remediation of the potential environmental impacts (e.g. water treatment and ecological restoration) can have positive net effects on the environment [23]. Mine abandonment, decommissioning and repurposing may positively and negatively affect social impacts. Examples of negative impacts include loss of jobs and local identities [24], while positive impacts may include opportunities for new economic activities [25], for example through repurposing of mines into tourist attractions.

### Mitigation measures

‘Mitigation measures’ (as they are commonly described in the impact assessment literature) are implemented to avoid, eliminate, reduce, control or compensate for the negative effects of an intervention and ameliorate the local impacts [23]. Typically, these measures should be considered and described in environmental and social impact assessments (EIAs and SIAs) conducted before major activities, such as resource extraction, begin [26–28]. If there will be a significant impact, mitigation measures are required by law in most countries to be implemented and monitored. Mitigation of negative environmental effects in one system (e.g. water or soil) can influence other systems in a positive or negative manner, such as the wellbeing of local communities [23]. A wide range of technologies have been implemented in the treatment of contaminated waters (e.g. constructed wetlands [29], reactive barriers treating groundwater [30], conventional wastewater treatment plants). Phytoremediation of contaminated land is also an active research area [31].

Mitigation measures designed for the alleviation of negative impacts of mining on social and environmental systems may not always be effective and may have undesired and unintended consequences, particularly in the long-term and across diverse systems (e.g. the intersection between environmental and social): for example, a mitigation measure designed to effect an environmental change may have unintended knock-on social impacts. To date, there appears to be little research on the effectiveness of mitigation measures applied to mining projects to achieve the desired mitigation outcome, and we are not aware of any synthesis of the effectiveness of metal mining oriented mitigation measures that considers impacts on both society and the environment.

### Mining in the Arctic and boreal regions

The Arctic and boreal regions are particularly sensitive to the effects of mining and mining-related activities [32, 33], both on social and environmental systems. However, the Arctic is home to substantial mineral resources [34, 35] and has been the focus of interest and resource extraction for centuries. These activities significantly increased during the early 20th Century and there has been an intensification of interest in exploration and exploitation in recent years to meet a growing global metal demand. Given the region’s geological features and expectations of growing global demand for metals, resource extraction already dominates discourse on development here and is likely to continue to do so for the near future. As of 2015, there were some 373 mineral mines across Alaska, Canada, Greenland, Iceland, The Faroes, Norway (including Svalbard), Sweden, Finland and Russia (see Table 1), with the top five minerals being gold, iron, copper, nickel and zinc [36]. Mining in the Arctic has generally intensified: for example, whilst the number of mines in Sweden has decreased substantially over the last 100 years, from a peak of  > 260 in 1917 to just 12 in 2019, the volume of production has conversely increased from < 5 m tonnes to 86.5 m tonnes over the same period [37].

**Table 1:** List of minerals mined across Arctic and boreal countries (Alaska (US), Canada, Greenland, Iceland, The Faroes, Norway (including Svalbard), Sweden, Finland and Russia) and the number of mines according to a 2015 survey [36]

Main metal mined	Number of mines
Gold	144
Iron	58
Copper	45
Nickel	39
Zinc	27
Diamonds	15
Uranium	8
Potash	7
Silver	6
Molybdenum	4
Lead	4
Chromium	3
Titanium	2
Tin	2
Tungsten	1
Palladium	1
Nobelium	1
Platinum	1
Lithium	1
Rare earth oxides	1
Antimony	1
Manganese	1
Aluminium	1

There is little empirical research on the impacts of mining on environmental and social systems in the literature. For example, there is a dearth of evidence on these impacts on the Sami; a group of traditional people inhabiting a region spanning northern Norway, Sweden, Finland and Russia. Sami people are affected by a range of external pressures, one of which pertains to resource extraction and land rights, particularly in relation to nomadic reindeer herding. However, there is little published research on the topic [38]. Indigenous peoples reside in many other regions within Arctic and boreal ecosystems (e.g. the many Aboriginal communities in Canada [39]) and these systems have been shown to be equally limited in evidence on social impacts of resource extraction [40, 41]. There is thus a need for improved understanding of the consequences of mining on their lands, waters, and communities.

The evidence base for research on the environmental and social impacts of mining has grown over recent years. However, despite the clear importance of this topic, there has been little rigorous synthesis of research knowledge in Arctic and boreal regions (although see [42] for a review with high susceptibility to bias on the topic). This lack of rigorous synthesis represents a significant knowledge gap in the face of the continued promotion and expansion of resource extraction in the region. There is thus an urgent need for transparent and robust approaches to collate and describe the nature of research evidence on the environmental and social impacts of mining and its mitigation measures.

### Stakeholder and rightsholder Engagement

This systematic map forms part of a broader knowledge synthesis project called 3MK (Mapping the impacts of Mining using Multiple Knowledges, https://osf.io/cvh3u). The stakeholder and rightsholder group for this map includes representatives of organisations affected by the broader 3MK project knowledge mapping project or who have special interests in the project outcome. We define stakeholders and rightsholders here as all individuals or organisations that might be affected by the systematic map work or its findings [43, 44], and thus broadly includes researchers and the Working and Advisory Group for this project.

The systematic map stakeholders and rightsholders and those of the broader 3MK project have an intentionally Scandinavian focus as a result of our research interests and funding. However, evidence on base metal mining impacts in Arctic and Boreal regions was strongly felt to be globally relevant to the review, and as such, the geographic scope of evidence considered was pan-Arctic/Boreal. Some members of the team are based in Canada and the representation of authors on this review was therefore felt to be beneficial in terms of identifying grey literature sources and the search string (see below).

Invitations to be included in this group were based on an initial stakeholder/rightsholder mapping process and soliciting expressions of interest (see Stakeholder Engagement Methodology Document, https://osf.io/cvh3u). This group included government ministries and agencies such as the Ministry of Enterprise and Innovation, the Mineral Inspectorate (Bergstaten) and County Administrative Boards, the mining industries’ branch organisation (Svemin) and individual companies such as LKAB Minerals and Boliden AB, Sami organisations, including the Sami Parliament, related research projects, and representatives of international assessment processes, such as activities within the Arctic Council. Stakeholders and rightsholders were invited to a specific meeting (held at Stockholm Environment Institute in September 2018) to help refine the scope, define the key elements of the review question, finalise a search strategy, and suggest sources of evidence, and also to subsequently provide comments on the structure of the final protocol [45].

### Objective of the review

The broader 3MK project aims to develop a multiple evidence base methodology [46] combining systematic review approaches with documentation of Indigenous and local knowledge and to apply this approach in a study of the impacts of metal mining and impacts of mitigation measures. This systematic map was conducted in order to answer the question:

#### What research evidence exists on the impacts of metal mining and its mitigation measures on social and environmental systems in Arctic and boreal regions?

### Definition of the question components

The review question has the following key elements:

*Population* Social, technological (i.e. industrial contexts, heavily altered environments, etc.) and environmental systems in circumpolar Arctic and boreal regions.

*Intervention/exposure* Impacts (direct and indirect, positive and negative) associated with metal mining (for gold, iron, copper, nickel, zinc, silver, molybdenum and lead) or its mitigation measures. We focus on these metals as they represent approximately 88% of Arctic and boreal mines (according to relevant country operating mine data from 2015 [36]), and contain the 5 most commonly mined minerals in the region (gold, iron, copper, nickel and zinc). Furthermore, these minerals include all metals mined within Sweden, the scope of a related workstream within the broader 3MK project (https://osf.io/cvh3u).

*Comparator:* For quantitative research; the absence of metal mining or metal mining mitigation measures—either prior to an activity or in an independent, controlled location lacking such impacts. Additionally, alternative mining systems are suitable comparators. For qualitative research; comparators are typically implicit, if present, and are thus not required.

*Outcome* Any and all outcomes observed in social and environmental systems described in the literature were iteratively identified and catalogued. Measured outcomes should be linkable to mining activities or their mitigation measures in the Arctic, irrespective of the scale of the intervention/exposure.

*Data type* Both quantitative and qualitative research were included.

## Methods

The review follows the Collaboration for Environmental Evidence (CEE) Guidelines and Standards for Evidence Synthesis in Environmental Management [47] and it conforms to ROSES reporting standards [48] (see Additional File 1). The review was conducted according to the published protocol [45].

### Deviations from the protocol

We made the following deviations from our published protocol. Several bibliography databases could not be used as planned:

- Worldwide Political Science Abstracts (via Proquest)—subscription lapsed
- JSTOR—an update to the search functionality meant that complex search strings are no longer accepted and a basic search was not functional at the time of conduct (error: ‘string too long’)
- AGRIS—advanced search and export not functional at the time of search conduct
- CAB Abstracts (via CAB Direct)—search facility not functional at the time of search conduct

Of the 104 proposed organisational websites, 19 sites could be searched: 9 were duplicate sub-organisations already included in other websites, 9 websites were unavailable at the time of searching, and 1 website could not be searched because of the departure of a Russian speaking colleague (*n* = 1). We chose not to search Google Scholar because of limited resources. Similarly, due to the COVID-19 pandemic and staff personal circumstances, we did not have resources available to perform a search update prior to publication.

### Search for articles

**Bibliographic databases** Searches for relevant research evidence were conducted between 27 and 29th November 2018 using the 21 bibliographic databases shown in Table 2.

**Table 2:** List of bibliographic databases and platforms searched for evidence along with the platform and subscription through which they were accessed

Database/index	Platform/provider	Date ranges	Date searched	Results	Search record link
Academic search premier	EBSCOhost	1956-	27/11/2018	5753	https://doi.org/10.1079/searchRxiv.20210350283
Agricola	National agricultural library	1700-	28/11/2018	7	https://doi.org/10.1079/searchRxiv.20210355628
Aquatic sciences and fisheries abstracts	ProQuest	1971-	28/11/2018	6835	https://doi.org/10.1079/searchRxiv.20210350284
DOAJ	DOAJ	2003-	29/11/2018	47	https://doi.org/10.1079/searchRxiv.20210355629
EconLit	EBSCOhost	1969-	29/11/2018	697	https://doi.org/10.1079/searchRxiv.20210350285
Greenfile	EBSCOhost	Unclear	29/11/2018	877	https://doi.org/10.1079/searchRxiv.20210350286
International Bibliography of the Social Sciences (IBSS)	ProQuest	1951-	29/11/2018	1238	https://doi.org/10.1079/searchRxiv.20210350287
MEDLINE	Web of science	1950-	28/11/2018	1869	https://doi.org/10.1079/searchRxiv.20210350288
ProQuest dissertations and theses	ProQuest	1861-	29/11/2018	33	https://doi.org/10.1079/searchRxiv.20210355630
PsycINFO	ProQuest	1806-	29/11/2018	213	https://doi.org/10.1079/searchRxiv.20210350289
Russian science citation index	Web of science	2005-	28/11/2018	704	https://doi.org/10.1079/searchRxiv.20210350290
Scopus	Scopus	1966-	29/11/2018	15609	https://doi.org/10.1079/searchRxiv.20210350291
Sociological abstracts	ProQuest	1952-	29/11/2018	266	https://doi.org/10.1079/searchRxiv.20210350292
Science citation index expanded	Web of science	1945–2018	28/11/2018	11518	https://doi.org/10.1079/searchRxiv.20210355623
Social sciences citation index	Web of science	1956–2018			https://doi.org/10.1079/searchRxiv.20210355622
Arts and humanities citation index	Web of science	1975–2018			https://doi.org/10.1079/searchRxiv.20210355624
Conference proceedings citation index-science	Web of science	1990–2018			https://doi.org/10.1079/searchRxiv.20210355625
Conference proceedings citation index-social science and humanities	Web of science	1990–2018			https://doi.org/10.1079/searchRxiv.20210355626
Emerging sources citation index	Web of science	2015–2018			https://doi.org/10.1079/searchRxiv.20210355627
DART-Europe E-theses portal	DART-Europe	Unclear	29/11/2018	0	https://doi.org/10.1079/searchRxiv.20210355631
EThOS	British library	Unclear	29/11/2018	0	https://doi.org/10.1079/searchRxiv.20210355632

The following Boolean search string formed the basis of searches:

(mine OR mined OR mining OR mines OR (extract* AND resource*) OR (extract* AND industr*) OR (extract* AND (mineral OR minerals)))

*AND*

(metal* OR iron OR copper OR nickel* OR lead OR zinc OR hematite OR haematite OR magnetite OR chalcopyrite OR digenite OR azurite OR malachite OR chrysocolla OR atacamite OR ore OR mineral* OR tailing OR pyrite OR ferric OR ferrous OR goethite OR limonite OR siderite OR ochre OR cupric OR chalcocite OR tenorite OR cuprite OR bornite OR covellite OR tetrahedrite OR tennantite OR pentlandite OR millerite OR galena OR kamacite OR taenite OR laterite OR garnierite OR boulangerite OR anglesite OR cerussite OR pyromorphite OR calamine OR smithsonite OR sphalerite OR hemimorphite OR wurzite OR hydrozincite OR pb OR zn OR cu OR ni OR fe OR gold OR au OR silver OR ag OR argenite OR chlorargyrite OR galena OR calaverite OR sylvanite OR nagyagite OR petzite OR krennerite OR molybden* OR wulfenite OR powellite OR mo)

*AND*

(*arctic OR boreal OR boreo* OR *polar OR "snow forest" OR tundra OR taiga OR alaska OR canada OR russia OR sweden OR norway OR finland OR greenland OR iceland OR faroe OR canadian OR swedish OR norwegian OR russian OR icelandic OR subarctic OR "northern latitude" OR "high latitude" OR yukon OR nunavut OR quebec OR "northwest territories" OR newfoundland OR labrador OR alaskan OR sibera OR ural OR volga OR caucasus OR lapland OR lappland OR norrbotten OR västerbotton OR ångermanland OR jämtland OR medelpad OR härjedalen OR hälsingland OR dalarna OR gästrikland OR uppland OR västmanland OR värmland OR ostrobothnia OR kainuu OR karelia OR savonia OR pirkanmaa OR satakunta OR tavastia OR kymenlaakso OR uusimaa OR åland OR Trøndelag OR "kalaallit nunaat" OR avannaata OR qeqertalik OR qeqqata OR sermersooq OR kujalleq OR aleutia* OR "british columbia" OR alberta OR saskatchewan OR manitoba OR "new brunswick" OR "prince rupert island" OR ontario OR "nova scotia" OR "north* europe" OR meadowbank OR ekati OR Meadowbank OR bellekeno OR "Keno Hill" OR Minto OR Tom OR Raglan OR Wolverine OR "Pine point" OR "Red Chris" OR Granduc OR Thompson OR Birchtree OR Seabee OR "Voisey's Bay" OR DSO OR "james mine" OR Schefferville OR "Flin Flon" OR "Triple Seven" OR "Snow lake" OR Huckleberry OR Carol OR Scully OR Wabush OR "Bloom lake" OR "mont wright" OR Eleonore OR "qr gold" OR Musselwhite OR Gibraltar OR "mcleese lake" OR Campbell OR "red lake" OR "rice lake" OR Bralorne-Pioneer OR "new afton" OR "highland valley" OR "detour lake" OR Ming OR rambler OR "pine cove" OR "corner bay" OR "bachelor lake" OR "myra falls" OR "casa berardi" OR Vezza OR "copper mountain" OR similco OR Langlois OR grevet OR Trail OR "duck pond" OR Hemlo OR Williams OR "kidd creek" OR "david bell" OR Hislop OR "bell creek" OR Beaufor OR "black fox" OR glimmer OR Holloway OR Holt OR Porcupine OR "fabie bay" OR "doyon division" OR LaRonde OR Lapa OR Mishi OR Macassa OR Sigma OR Lamaque OR Goldex OR "eagle river" OR Young-Davidson OR "Halfmile lake" OR Coleman OR "nickel rim" OR Fraser OR "McCreedy west" OR Sudbury OR Garson OR Stobie OR "Copper cliff" OR Manitoba OR ontario OR Creighton OR Gertrude OR Ellen OR Lockerby OR Totten OR Dufferin OR Bingo OR "Bonanza ledge" OR Bracemac-McLeod OR Cochenour OR "EP gold" OR Nunavik OR "fire lake" OR "hudson bay" OR Island OR "lac herbin" OR "lalor lake" OR Malartic OR Morrison OR "mount milligan" OR Phoenix OR rubicon OR "reed lake" OR Shakespeare OR "timmins west" OR McGarry OR Westwood-Mooshla OR Yellowjacket OR Alexo OR Monique OR "yellow giant" OR Kittilä OR Kevitsa OR Pahtavaara OR Hannukainen OR Laiva OR Talvivaara OR Kokkola OR Hitura OR Pyhäsalmi OR Pampalo OR Kylylahti OR Orivesi OR Kutemajärvi OR Vammala OR Harjavalta OR Jokisivu OR Sydvaranger OR Rana OR Odda OR Oktyabrsky OR Talnakhskoye OR Taimyrsky OR Komsomolsky OR Mayak OR Kaula-Kotselvaara OR Kola OR Zapolyarnoye OR Zapolyarny OR Ametistovoe OR "Medvezhy rucheu" OR Norilsk-1 OR Skalisty OR Zhdanovskoye OR Mayskoye OR Olenegorsky OR Olenegorsky OR "15th anniversary of october" OR Kovdorsky OR Kupol OR Korpanga OR Kostomuksha OR Kubaka OR Arylakh OR Lunnoye OR Susuman OR Berelekh OR Dukat OR Omsukchan OR Julietta OR Natalka OR Olimpiada OR Sovetskoye OR Blagodatnoye OR Khakanja OR Tarnjerskoye OR Severny OR Severopeschanskoye OR Vorontsovskoye OR "Golets Vysochaishy" OR Kuranakh OR Gusevogorskoye OR Pervenets OR Goroblagodatsky OR Bereznyakovskoye OR Gorevsky OR Vysokogorsky OR Rudnogorsky OR Pervouralskoye OR Berezovskoye OR Tatianinsky OR Korshunovsky OR Safyanovskaya OR Cheremshansky OR Irokinda OR Aginskoye OR Bakalskoye OR Talgansky OR Berezitovy OR Svetlinsky OR Uchalinsky OR Uchalinsky OR Mulginskoye OR Burlukskoye OR Chebachje OR Pioneer OR Mnogovershinnoye OR Abakansky OR Aleksandrinsk OR Pokrovskiy OR Teysky OR Sheregeshsky OR Ozernoye OR Tashtagolsky OR Sibay OR Murzinskoye-1 OR Zun-Holba OR Mikhailovsky OR Asacha OR Novoshirokinskoye OR Oktyabrsky OR Verninsky OR Kyzyl-Tashtyg OR Rubtsovsk OR Osenneye OR Gaisky OR Stepnoy OR Korbalikhinsky OR Stoylensky OR Zarechenskoye OR Lebedinsky OR Stoylo-Lebedinskoye OR Letneye OR Dzhusinskoye OR Kimkanskoye OR Dalpolimetal OR Urupsky OR Sadonsk OR Albazino OR AldGold OR Amazarkan OR "Lena artelj" OR "Tyva artelj" OR Avlayakan OR Albyn OR Belaya OR Birkachan OR Buryatzoloto OR Eldorado OR Festivalnoye OR Fevralskoye OR Goltsovoye OR Gubkin OR Karalveem OR Kirovogorsky OR Kochkarskoye OR Komsomolsky OR Kamaganskoye OR Kazsky OR Kommunarovsky OR Lenzoloto OR "Maly kuybas" OR Malomir OR Mauksky OR Mikheevskoye OR Mezhsopochnoye OR "Novogodnee monto" OR Odinochnaya OR Podotvalnoye OR Pogromnoye OR Samolazovskoye OR Savkino OR Shemurskoye OR Sideritovaya OR Yuzhno-Kirovskoye OR "Sopka Kvartsevaya" OR Sosnovsky OR Tabornoye OR Tardan OR Titimukhta OR Tsokol OR Uzelginsky OR Vasilevsky OR Valunistoye OR Vysokogorsky OR Vysokogorsky OR Vetrensky OR Yakutskoye OR Yubileynoye OR Baumansky OR Degtyarskoye OR Delbe OR Dvoynoye OR Estyuninskaya OR Garbuzovskoye OR Kanavnoye OR Konevinskoye OR Korolevsky OR "South kurasan" OR "West kurasan" OR Kamenskoye OR Krasnokamenskoye OR Lunnoye OR Molodyozhny OR Nikolaevsky OR Nizhneyakokitskoye OR Odolgo OR Olenje OR Olenegorsky OR Ozerny OR Partizanskoye OR Serovsky OR Severnoye OR Severo-Zapadny OR Sibir-Polimetally OR Vesely OR Solcocon OR Svetly OR Shanuch OR Verkhneye OR Yurjevskoye OR Yuzhnoye OR Kiruna OR Kaunisvaara OR Tapuli OR Malmberget OR Salmijärvi OR Aitik OR Kristineberg OR Maurliden OR Renström OR Boliden OR Björkdal OR Kankberg OR Svartliden OR Garpenberg OR Dannemora OR Lovisa OR Zinkgruvan OR Gruvberget OR "red dog" OR "fort knox" OR Pogo OR "stone boy" OR Kensington OR "greens creek" OR "Kelex" OR "Sinyukhinskoye" OR Endako OR Kitsault OR Zhireken OR Sorskoye)

The search string was developed based on intervention terms (mining) combined with metal terms and location terms. Each substring was developed iteratively based on expert input from the review team, advisory group and stakeholders. Location terms were supplemented by collating names for all known mines in boreal and Arctic regions.

For details of the search string adaptations used in each resource, see the searchRxiv search history records detailed in Table 2. Searches of bibliographic databases were performed only in English, since in each case the bibliographic data were translated to English prior to indexing, meaning that full texts in non-English language would be found this way. Specialist searches (see below) were performed in other languages.

**Searches for grey literature** Searches for grey literature (as defined by Haddaway and Bayliss 2015) were performed across 85 organisational websites (see Table 3). These searches involved manual screening of each website for a ‘publications’ section, followed by searching using basic search terms if a search facility was present. For all English-language websites, searches were performed in English using the following core terms where search functionality allowed: mining impacts; mining effects; mining mitigation; social impacts mine; environmental impacts mine. For 6 websites with non-English language content (Finnish Game and Fisheries Research Institute; Greenland Institute of Natural Resources; Ministry of Natural Resources of the Russian Federation; Norwegian Directorate for Nature Management; Norwegian Institute for Nature Research (NINA); Finnish Sami Parliament; Norwegian Sami Parliament), searches were performed in Finnish (metallien louhinnan vaikutukset; metallien kaivostoiminta; kaivostoiminta; kaivannaisteollisuus; kaivos), and Danish (påvirkning virkning af metalminedrift; metalminedrift; minedrift) and Norwegian (støt effekter på gruvedrift av metal; metall gruvedrift; gruvedrift) as appropriate.

**Table 3:** Organisations whose websites were searched for relevant evidence

Organisation	URL
Alaska department of natural resources	http://dnr.alaska.gov
Alaska Division of Geological and Geophysical Surveys	http://dggs.alaska.gov/
All-Russian Geological Research Institute. A.P. Karpinsky	http://www.vsegei.ru/
Arctic Centre (University of Lapland)	http://www.arcticcentre.org
Arctic Council	http://www.arctic-council.org
Arctic Health	https://arctichealth.nlm.nih.gov
Arctic Health (Finland)	http://www.oulu.fi/arctichealth/
Arctic Monitoring and Assessment Programme (AMAP)	https://www.amap.no/
Arctic Research Centre	http://arctic.au.dk/
ArcticNet	http://www.arcticnet.ulaval.ca/
Aurora Research Institute	http://nwtresearch.com/research-projects/information-technology/arctic-collaborative-environment
Bureau of Land Management, US Dept. of the Interior	http://www.blm.gov
Canadian Institute of Health	http://www.cihr-irsc.gc.ca/e/193.html
Centre for Indigenous Peoples' Nutrition and Environment (CINE)	http://www.mcgill.ca/cine/
Centre for Saami Health Research	http://en.uit.no/ansatte/organisasjon/hjem?p_menu=42374&p_dimension_id=88182
Conservation of Arctic Flora and Fauna (CAFF)	http://www.caff.is
Copper Alliance—The International Copper Association	http://copperalliance.org/
Cultural Survival	https://www.culturalsurvival.org/
Environment and Climate Change Canada	http://www.ec.gc.ca
European Commission	http://ec.europa.eu/
European Environment Agency	http://www.eea.europa.eu/
Faroese Geological Survey: Jarðfeingi	http://jf.fo/en/
Federal Agency for Mineral Resources	http://government.ru/en/department/53/
Finnish Environment Institute	http://www.environment.fi/
Finnish Game and Fisheries Research Institute	http://www.rktl.fi
Fridtjof Nansen Institute	https://www.fni.no/
Geological Survey of Denmark and Greenland (GEUS)	http://www.eng.geus.dk/
Geological Survey of Finland	http://en.gtk.fi/
Geological survey of Norway	https://www.ngu.no/en
Greenland Institute for Circumpolar Health Research	http://www.pi.gl/da
Greenland Institute of Natural Resources	http://www.natur.gl
Greenland Institute of Natural Resources	https://education.uarctic.org/universities/greenland/23857/greenland-institute-of-natural-resources
GRID Arendal	http://www.grida.no
Institute of Arctic Biology	http://www.iab.uaf.edu/
International Arctic Research Center (IARC)	http://www.iarc.uaf.edu/
International Arctic Social Sciences Association (IASSA)	http://www.iassa.org/
International Copper Study Group	https://www.icsg.org/
International Iron Metallics Association	https://www.metallics.org/
International Lead and Zinc Study Group	http://www.ilzsg.org/static/home.aspx
International Lead Association	https://www.ila-lead.org/home
International Molybdenum Association (IMOA)	https://www.imoa.info/index.php
International Nickel Study Group	http://www.insg.org/
International Resource Panel	http://www.resourcepanel.org/
International Union for Conservation of Nature	http://www.iucn.org
International Zinc Association	https://www.zinc.org/about/
Isaaffik	http://www.isaaffik.org/
Luleå University of Technology	https://www.ltu.se/?l=en
Ministry of Natural Resources of the Russian Federation	http://www.mnr.gov.ru
Natural Resources Canada	http://www.nrcan.gc.ca
NGO Mining Working Group	https://miningwg.com/
Nickel Institute	https://www.nickelinstitute.org/
Nordic Council of Ministers	http://www.norden.org
Northern Research Institute (NORUT)	http://www.norut.no
Norwegian Directorate for Nature Management	http://www.dirnat.no
Norwegian Environment Agency	http://www.miljodirektoratet.no/en/
Norwegian Institute for Nature Research (NINA)	http://www.nina.no
Norwegian Institute for Water Research (NIVA)	http://www.niva.no
Norwegian Polar Institute	http://www.npolar.no
Nunavut Research Institute	http://www.nri.nu.ca/
Polar Environmental Atmospheric Research Laboratory (PEARL)	https://eu-interact.org/field-sites/polar-environment-atmospheric-research-laboratory-pearl/
Polar Research Board	http://dels.nas.edu/prb
RAND Corporation	https://www.rand.org/topics/russia.html
Resource Extraction and Sustainable Arctic Communities project (REXSAC)	https://www.rexsac.org/
Russian Regional Environmental Centre	http://www.rusrec.ru
Saami Council	https://www.saamicouncil.net/
Sámediggi (Finnish Sami Parliament)	http://www.samediggi.fi
Sámediggi (Norwegian Sami Parliament)	http://www.sametinget.no
Stockholm Environment Institute	http://www.sei.org/
Strategic innovation programme for the Swedish mining and metal producing industry	https://www.sipstrim.se/
Swedish Agency for Marine and Water Management	http://www.havochvatten.se
Swedish Environmental Protection Agency (SEPA)	http://www.swedishepa.se/
Swedish Geological Survey	https://www.sgu.se/en/
Swedish University of Agricultural Sciences (SLU)	http://www.slu.se
The Arctic Institute: Center for Circumpolar Security Studies	http://www.thearcticinstitute.org/
The European Network for Sustainable Quarrying and Mining	https://ensqm.weebly.com/
The World Bank	https://www.worldbank.org/
Thule Institute	http://www.oulu.fi/thuleinstitute/
United Nations Environment Programme	http://www.unep.org
United States Environmental Protection Agency	http://www3.epa.gov/
United States Fish and Wildlife Service	http://www.fws.gov
University of Alaska Anchorage	http://www.uaa.alaska.edu
University of Eastern Finland	http://www.uef.fi/en/etusivu
Uppsala University Department of Earth Sciences	https://www.geo.uu.se/research/geophysics/ongoing-research/mineral-exploration/
World Gold Council	https://www.gold.org/
World Steel Association	https://www.worldsteel.org/
Yukon Research Centre	http://www.yukoncollege.yk.ca/research

**Supplementary searches** In addition, we (Adrienne Smith [AS] and Brooke Etherington [BE]) hand searched reference sections of relevant included articles and 47 randomly selected relevant literature reviews which accounted for 30% of the relevant reviews identified in the searching (see Additional File 2).

### Estimating the comprehensiveness of the search

A set of 49 articles known to be relevant were provided by the review team; the benchmark list (see Additional File 3). During scoping and development of the search string, the bibliographic database search results were checked to ascertain whether any of these studies were not found. We found 45 of the 49 benchmark based on search term presence in titles, abstracts and keywords (see Additional File 4). Four articles were not found because they lacked abstracts and/or keywords. This was deemed to be an appropriate retrieval level considering the complementary searching outlined above.

### Article screening and study eligibility criteria

All articles were screened according to the established eligibility criteria developed in the protocol [45]. All inclusion criteria were used at title, abstract, and full text screening. However, *data type* and *comparator* were not considered useful at title and abstract screening since this information is often not well-reported in these fields.

### Eligibility criteria

The following criteria were used to assess relevance (eligibility) of studies identified through searching.

**Eligible population** We included social, technological and environmental systems in Arctic and boreal regions based on political boundaries as follows (this encompasses various definitions of boreal zones, rather than any one specific definition for comprehensiveness and ease of understanding): Canada, USA (Alaska), Greenland, Iceland, the Faroe Islands, Norway (including Svalbard), Sweden, Finland, and Russia.

**Eligible intervention/exposure** We included all impacts (positive, negative, direct and indirect) associated with any aspect of metal mining and its mitigation measures. We included research pertaining to all stages of mining, from prospecting onwards as follows: prospecting, exploration, construction, operation, maintenance, expansion, abandonment, decommissioning, reopening and repurposing. Eligible mines included those of gold, iron, copper, nickel, zinc, silver, molybdenum and lead.

**Eligible comparator** For quantitative research; the absence of metal mining or metal mining mitigation measures—either prior to an activity or in an independent, controlled location lacking such impacts. For qualitative research; comparators are typically implicit, if present and thus were not required.

**Eligible outcome** Any and all outcomes (i.e. measured impacts) observed in social, technological and environmental systems were included.

**Eligible data type** We included quantitative, qualitative and mixed methods research.

**Eligible study type** We included both primary empirical research and secondary research (reviews were catalogued in a separate database). Modelling studies and commentaries were not included.

### Screening process

Articles found by searches in databases were combined and screened at three distinct stages: (1) title (2) title and abstract, and (3) full-text. Articles found by other means (i.e., organisational websites and review references) were screened at title and abstract and full-text but were not included in consistency checks.

Any doubt over the presence of a relevant inclusion criterion (or if information is absent) resulted in the articles being retained for assessment at a later stage.

Titles were screened by three reviewers (NRH, JJT, CA). This stage was not subject to consistency checking—it was conducted as a preliminary stage to remove all clearly irrelevant records that could be obviously discerned as ineligible based on titles. Records for which any doubt remained were included to be conservative.

Prior to screening abstracts, all reviewers conducted a consistency checking procedure in EPPI-Reviewer to ensure consistent and repeatable decisions were being made among reviewers in regards to which records were deemed eligible. A total of 1,655 abstracts were screened by 4 reviewers as follows. In the first round, the agreement level achieved was 369/510 abstracts (NRH vs CA). Following discussion, the second round increased slightly to 205/271 (NRH vs JJT) and 216/271 (CA vs JJT). A third round resulted in a 372/510 (NRH vs JJT), 384/510 (JJT vs CA), and 370/510 (NRH vs CA) agreement level. A final round indicated a further increase in agreement, with 292/364 agreements (NRH vs CA). At this point, we felt consistency was as high as possible, and a continued conservative approach was applied to screening of the remaining abstracts. Reviewers did not screen articles (at title and abstract or full-text) for which they were an author.

A consistency check was also conducted prior to screening articles at full-text on 69 articles. Full-texts were screened by four reviewers [NRH, JJT, AS and Amanda Jeansen (AJ)], with a first round of screening resulting in 18/30 article agreement across all four coders. Disagreements were discussed at length and related primarily to uncertainty in this round. Consistency checking on a further set of 39 articles, resulting in only 7 disagreements (κ = 0.62). Following this consistency checking, the remaining articles were split between the two reviewers (AS and AJ) and allowed to proceed. Any query made by a reviewer was discussed with the review team (NRH, JJT, AS and AJ) and a consensus decision made, and conferred to all reviewers. Lists of all articles excluded on the basis of full-text assessment with the reasons for exclusion, and unobtainable articles are provided in Additional File 5. A list of articles that could not be retrieved at full text is available in Additional File 6.

### Study validity assessment

No formal in-depth critical appraisal was made of study validity after their inclusion in the systematic map, since it is an optional part of systematic mapping methodology [49]. However, meta-data referring to study setting and design were extracted that could aid future study validity assessments and synthesis of studies on sub-topics of interest identified from our systematic map exercise. We have synthesised these variables along with other meta-data.

### Data coding strategy

Following full-text screening of articles, relevant studies were extracted from the included articles: where multiple studies were reported within one article they were entered as independent lines in the database. Here, we define a study to be an experiment or observation that was undertaken at a particular mine site, mitigation or ex-situ experiment with mine-specific treatments. Studies were separated by line if the intervention impacted different systems (populations) within the same article.

The following key data domains were identified through scoping activities and discussion with the review team and advisory group*:* (1) bibliographic information; (2) mine location and details (e.g., geographic location, metal extracted, type, stage, etc.); (3) broad objectives of the study; (4) study design and setting; (5) system affected; (6) impact/mitigation; (7) measured outcomes; (8) data type and location. Coding variables and meta-data within these domains were then compiled in a partly iterative process, expanding the range of options as they were encountered during scoping and extraction.

Where data are missing they were coded as ‘NR’ (not reported). Where coding is not applicable, ‘NA’ was recorded.

We adapted an outcome coding schema designed within an ongoing environmental and social impact assessment synthesis project [50]. The coding schema was developed to a small degree during trial data extraction. Our impact coding schema (including editions relative to the source) is outlined in Table 4.

**Table 4:** Coding impact schema

System affected	Component affected	Factor affected
Soil/geology	Soil surface	Structure
		Quality
		Relief
		Quantity^a^
	Soil (other)	Structure
		Quality
Water	Surface water	Surface drainage (run-off patterns)
		Quality
		Quantity
	Groundwater	Aquifers recharge
		Quality
		Quantity
	Ice	Ice^a^
	Sediment	Quality^a^
Air	Climate	Climate
	Atmosphere	Air quality
		Noise
		Vibrations^a^
		Light
Biodiversity	Flora	Habitat
		Species groups
		Individual species
		Species distribution
	Fauna	Habitat
		Species groups
		Individual species
		Species distribution
	Ecosystems	Quality
		Protected areas
Human environment	Landscapes	Scenic resources
		Change of land use
		Other qualities
	Economic	Jobs (new employment)
		Local business (e.g. local shops)
		Traditional livelihoods
		Property^a^
		Migration^a^
		Other^a^
	Service and infrastructure demand	Water
		Energy
		Waste
		Consumables/subsistence
		Trade
		Infrastructure
		Public health
		Municipal services
		Public safety (police) ^a^
		Other services^a^
	Culture/history	Cultural resources
		Archaeological sites
		Heritage
	Health/wellbeing	Health/safety^b^
		Education
		Recreation
		Env. justice
		Housing
		Other^a^

Adapted from [50]

^a^Indicates the addition of a code

^b^Indicates the merger of two related codes

To ensure that data were extracted in a consistent and repeatable manner, three reviewers (AS, JJT and NRH) piloted the extraction form by independently coding information from 10 articles at the beginning of the process. All disagreements were discussed, and additional, more detailed guidance was added to the extraction codebook to improve clarity. Coding and meta-data extraction then proceeded with one reviewer (AS). The finalised extraction form and codebook for the map (along with descriptions of each meta-data/coding field) is shown in Additional File 7.

### Data synthesis and presentation

We have summarised the review process in a ROSES flow diagram, using the ROSES flow diagram R package [51]. Our primary outputs are a searchable and filterable systematic map database provided as a CSV file (Additional File 8) along with an interactive evidence atlas (geographical information system) provided as a web-based app and downloadable HTML file (Additional File 9). All interactive files and visualisations are provided through the dedicated project website; https://3mkproject.github.io/.

We summarise the evidence base using bar plots, focusing first on the publications (e.g. article publication year), then on the study systems (i.e. the mines), the mitigation measures investigated, the study designs (e.g. data type), and the measured outcomes. We then describe the affected systems using our three-layer hierarchy (system, component, factor) to describe the social or environmental context that was reported to have been affected by the mining. We have visualised this using a bespoke radial bubble plot that clusters outcomes across the hierarchy and show the volume of evidence at each level. Finally, we have produced heat maps that visualise two coding factors (categorical variables) along with the volume of evidence found across combinations of levels of each factor. The interactive plots are available online (https://3mkproject.github.io/) and the code to produce them has been converted into an R script (Additional File 10). All data, code and functions are available on GitHub as an Open Source/Open Data resource here; https://github.com/nealhaddaway/3mk.

Knowledge gaps and clusters are highlighted by visually analysing cross tabulations and discussing candidate groups amongst the review team.

No team member was permitted to review their own work. Team members conducting screening, data extraction and coding were not publishing in this field.

## Review findings

### Review descriptive statistics

**Fig. 1:**
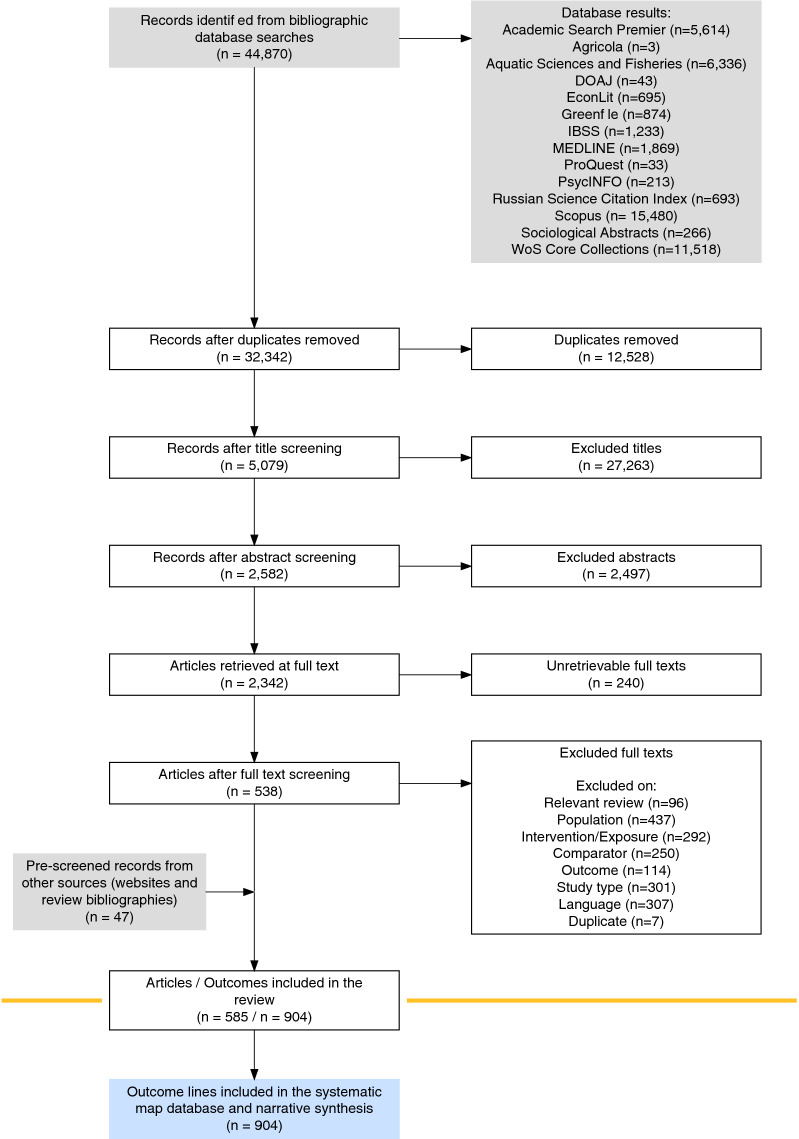
ROSES flowchart for the systematic map, showing the number of records retained at each stage of the review process. Produced using the R package ‘ROSES_flowchart’ [51]

The review process is depicted in a ROSES flow diagram (Fig. 1). We obtained a total of 44,870 records from bibliographic database searching. Following deduplication, 32,342 unique records were screened at title level and 5079 at abstract level. A total of 247 records could not be retrieved, leaving 2342 records screened at full text level. Following full text screening, 538 articles were retained. We included a further 47 articles from pre-screened resources (websites and review bibliographies). A final 585 articles were included in the map, corresponding to 902 outcome lines. We differentiate here between articles—the manuscripts in which data are presented—and outcome lines—the smallest independent data point in our map, corresponding to a measured outcome from a research article corresponding to a specific mine or mining area.

### The evidence base

**Fig. 2:**
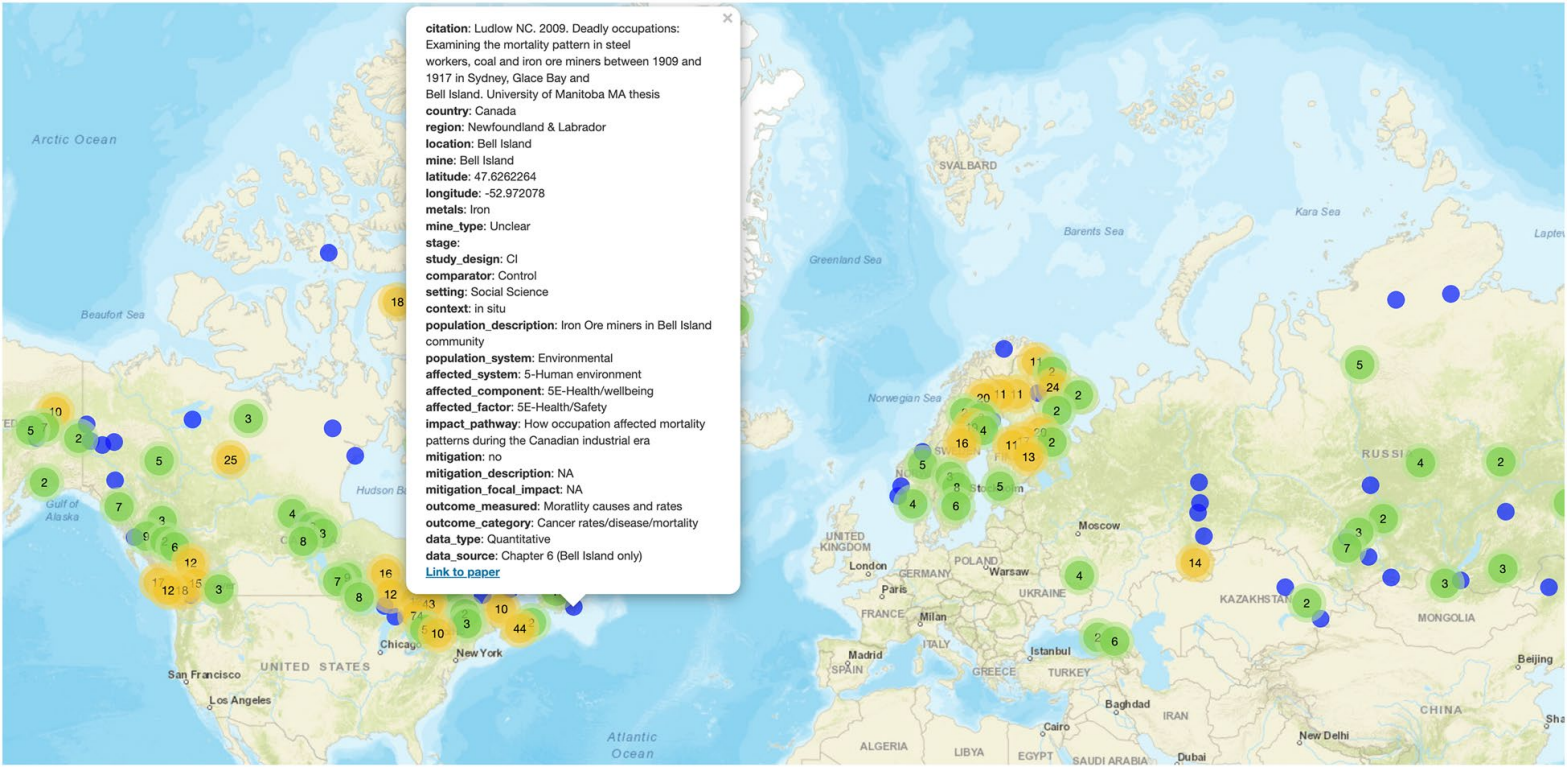
Screenshot of the interactive evidence atlas showing the location of all study systems in the 585 included studies across 902 total outcome measures. The popup contains descriptive meta-data and a link to the paper on Google Scholar. The interactive evidence atlas is available here: https://3mkproject.github.io/research.html

The interactive evidence atlas is available online (https://3mkproject.github.io/research.html) and as a downloadable HTML file (Additional File 9). We also present an exemplary screenshot in Fig. 2. The evidence atlas shows the location of each study system examined, displaying details of the system and the article describing it in a popup box that includes a hyperlink to the article on Google Scholar.

### The articles

**Fig. 3:**
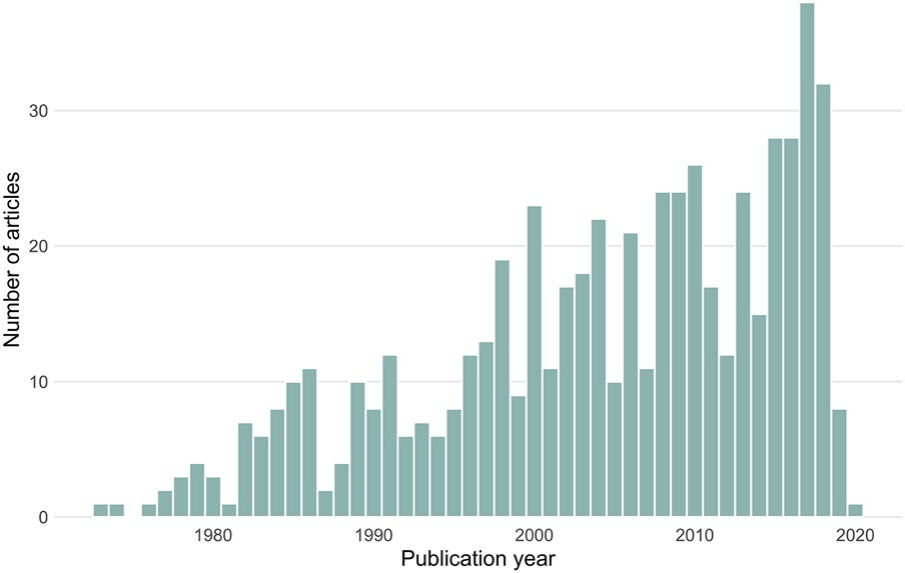
The publication years of articles included in the map

Figure 3 shows the publication dates of the included articles, suggesting a linear increase in the number of publications annually. This is in contrast to many other topics that seem to be experiencing a near exponential increase in published articles; perhaps because of the long and consistent history of mining relative to other topics, such as fossil fuel extraction. Although the environmental impacts of mining have a longer history [e.g. 52, the social impacts of mining is a fairly new research topic, and we may observe an increase in research attention overall in coming years.

**Fig. 4:**
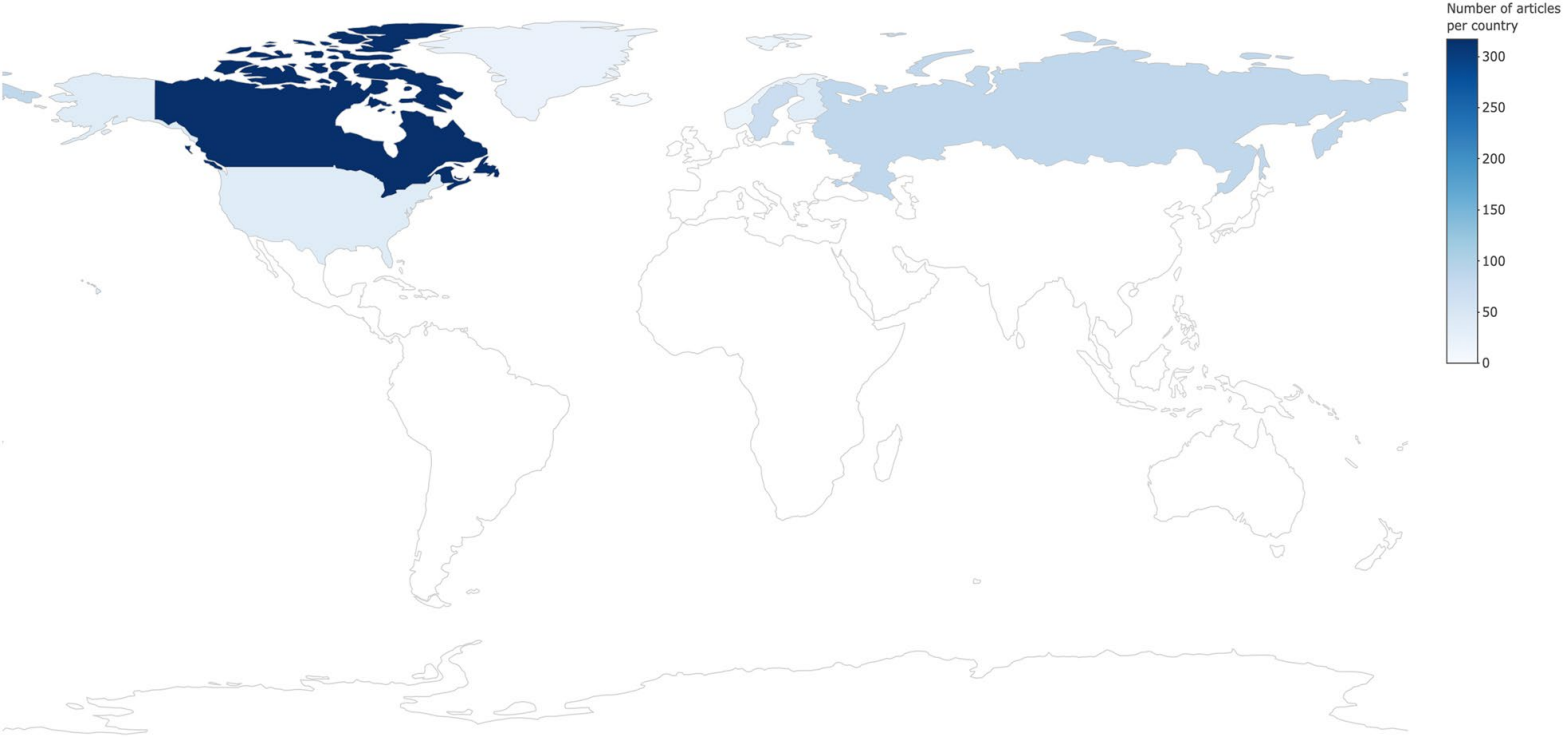
The number of articles in the map from across eligible Arctic and boreal countries

Figure 4 displays a choropleth for the number of articles included in the map from across eligible countries. The majority of articles focused on Canada (n = 317), followed by Russia (n = 84) and Sweden (n = 72). No research was identified from Iceland.

### Mines investigated

A total of 177 unique mines were described across the 585 articles: these are described in a database in Additional File 11. They were distributed across countries as follows: Canada, 97; Russia, 28; Sweden, 18; USA, 12; Finland, 9; Norway, 9; Greenland, 4; Iceland, 0.

**Fig. 5:**
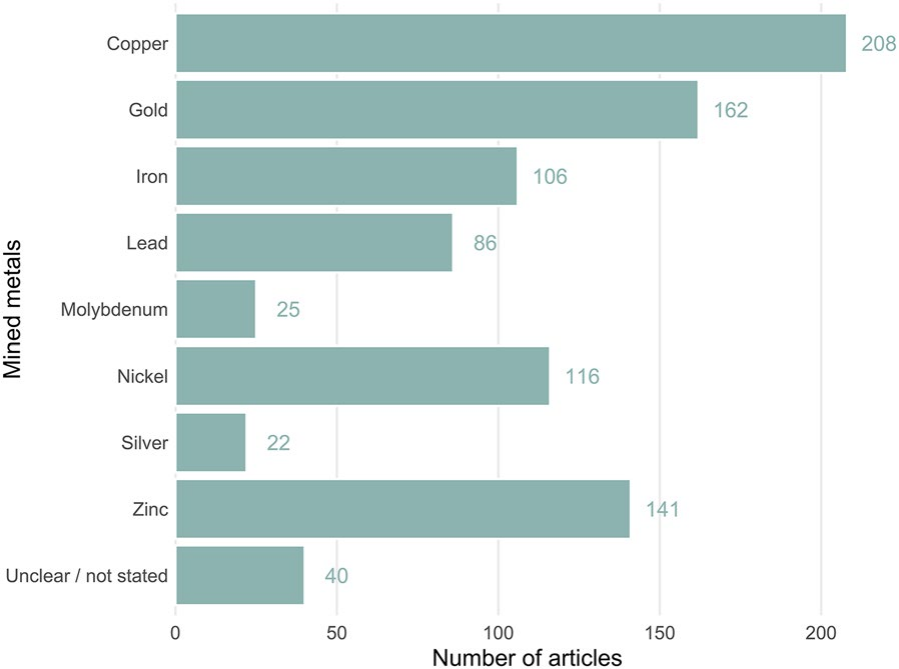
The number of articles in the map reporting different metals mined. Numbers are not mutually exclusive and include articles studying multi-ore mines

Copper was the most commonly reported metal (n = 208), followed by gold (n = 162) and zinc (n = 141) (see Fig. 5). The metals extracted were not stated in 32 articles. Some articles stated only the principal metal mined, whilst others reported all metals encountered and extracted—the data are therefore representative of the articles and may not reflect precisely the state of metals mined across the Arctic and boreal regions.

**Fig. 6:**
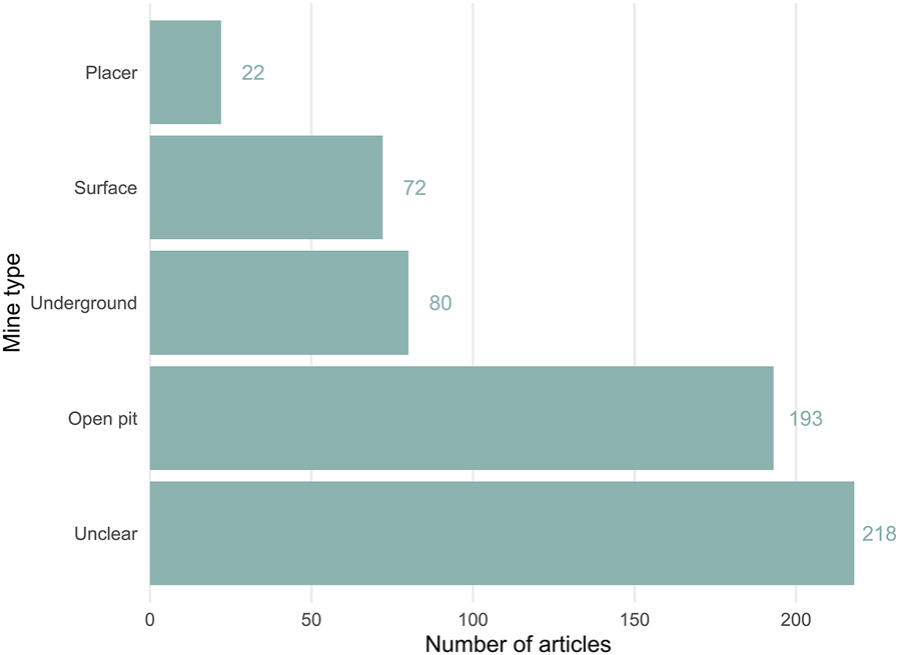
The number of articles in the map according to mine type reported

The most commonly reported mine type was open pit (n = 218), with less than half this number underground (n = 80) and surface (n = 72), and only 22 articles focused on placer mines (Fig. 6). Some 218 articles did not report the type of mine.

### Extraction stage

**Fig. 7:**
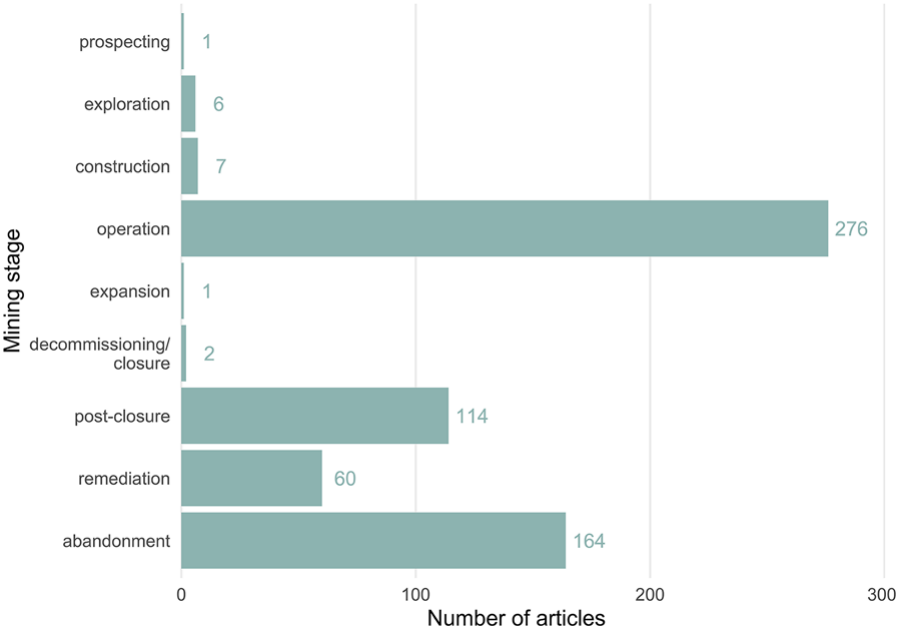
The number of articles in the map according to the extraction stage. Note some articles report multiple stages

The most commonly studied extraction stage was operation (n = 276), followed by abandonment (n = 164), post-closure (n = 114), and remediation (n = 60) (see Fig. 7). Prospecting, exploration, construction, expansion, and decommissioning/closure were studied far less frequently (n = 1–7).

**Fig. 8:**
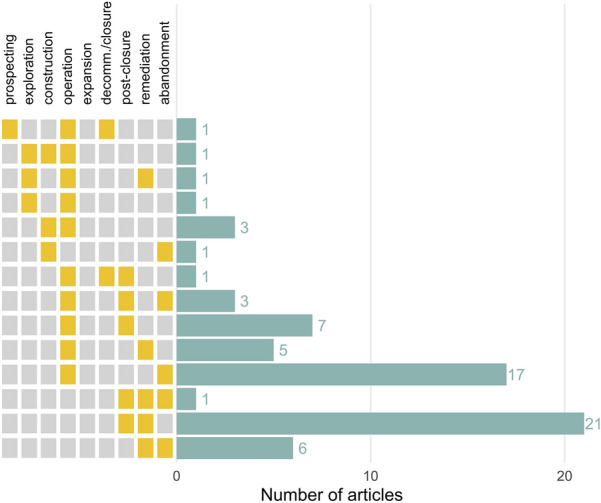
The number of articles in the map reporting multiple extraction stages, arranged by order of stages investigated. Yellow blocks indicate the stages were reported in the same article. Decomm./closure = decommissioning or closure

Figure 8 shows the articles reporting multiple extraction stages, demonstrating that the most commonly co-reported stages were post-closure and remediation (n = 21), followed by operation and abandonment (n = 17). Articles more commonly reported multiple stages following resource extraction activities (decommissioning/closure, post-closure, remediation and abandonment), than earlier stages. No articles reported more than three stages together.

### Mitigation measures

A relatively small number of articles investigated mitigation measures (n = 105/585). The systems, components and factors these mitigation measures were used to ameliorate are listed in Table 5. Groundwater quality was the most common factor mitigated (n = 57), followed by soil surface quality (n = 13) and flora species groups (n = 11). Full descriptions of the mitigation measures encountered are provided in the map database (Additional File 8).

**Table 5:** Systems, components and factors affected across the articles reporting mitigation measures

System > Component > Factor	Number of articles
Water > Groundwater > Quality	57
Soil/geology > Soil surface > Quality	13
Biodiversity > Flora > Species groups	11
Water > Surface water > Quality	5
Biodiversity > Flora > Individual species	4
Biodiversity > Fauna > Individual species	2
Biodiversity > Flora > Habitat	2
Water > Sediment > Quality	2
Air > Atmosphere > Vibrations	1
Biodiversity > Fauna > Species distribution	1
Biodiversity > Fauna > Species groups	1
Biodiversity > Flora > Species distribution	1
Human environment > Economic > Jobs (new employments)	1
Soil/geology > Soil surface > Structure	1
Water > Groundwater > Quantity	1
Water > Surface water > Quantity	1
Water > Surface water > Surface drainage (runoff patterns)	1

### Study design

**Fig. 9:**
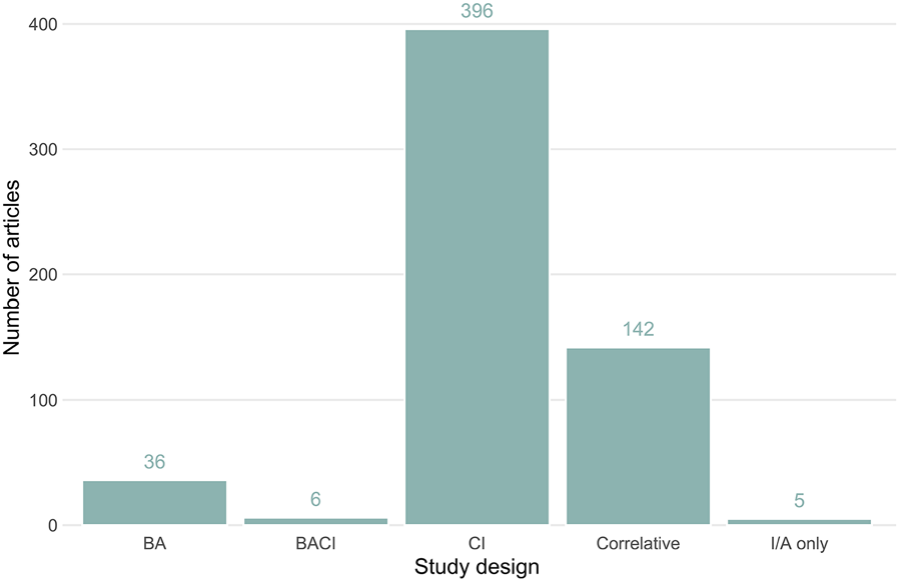
The number of articles across 5 study designs: *BA* before-after, *BACI* before-after-control-impacts, *CI* control-impact, *I/A only* impacts/affects only

Most articles in the map used a ‘control-impacts’ study design (n = 396), with a substantial number employing correlative designs (n = 142) (see Fig. 9). Only 5 articles examined just the impact/affected site with no real study design.

**Fig. 10:**
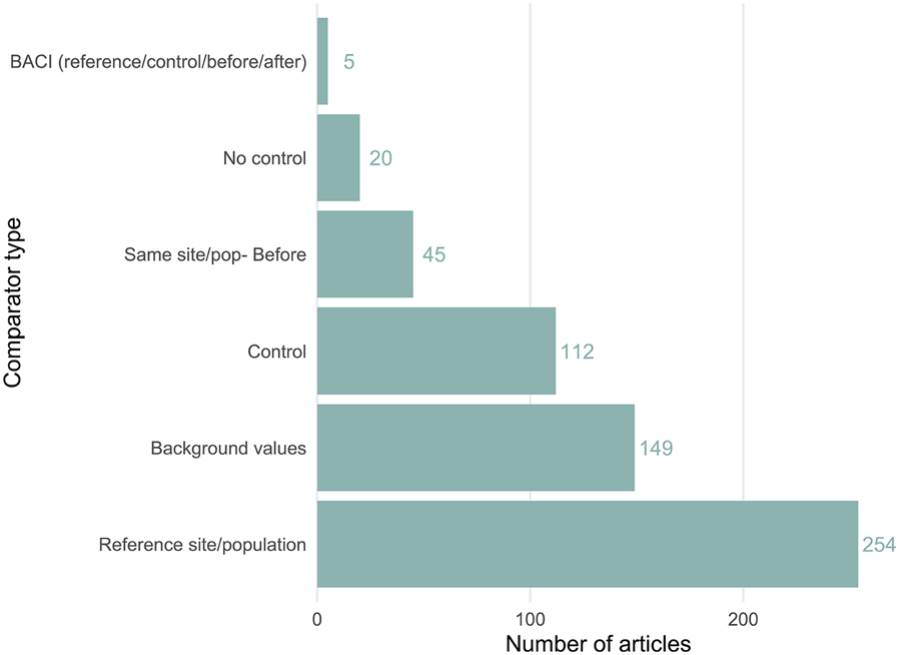
The number of articles using each type of comparator

Related to this, the precise type of comparator is elaborated in Fig. 10, demonstrating that the most common comparator was a reference site/population (n = 254), followed by background values (n = 149). Full BACI designs (before after control impacts) were least common (n = 5).

**Fig. 11:**
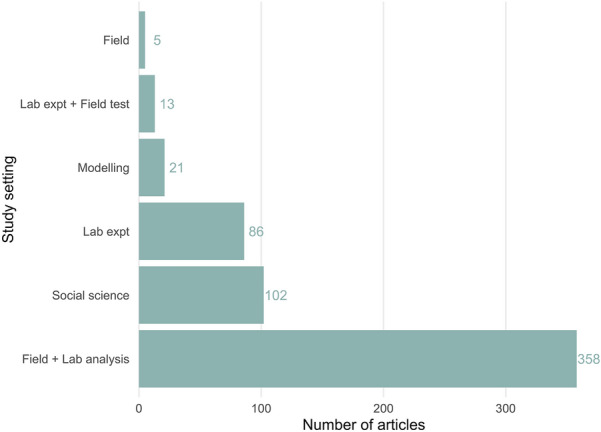
The number of articles reporting each type of study setting. Lab = laboratory; expt = experiment

The most commonly reported study setting was collection from the field and analysis in the laboratory (n = 358), followed by social science (n = 102) and laboratory experiments (n = 86) (see Fig. 11).

**Fig. 12:**
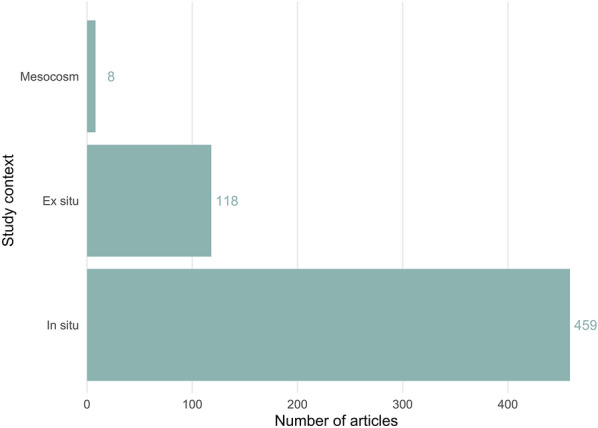
The number of articles reporting study contexts

Similarly, the context of the included articles’ studies was predominantly in situ (n = 459), with a smaller number using ex situ methods (n = 118) and very few employing mesocosms (n = 8) (see Fig. 12).

The great majority of articles reported quantitative data (n = 545), with a very small number collecting qualitative data (n = 40). Similarly, a total of 473 articles reported data from environmental systems, whilst 112 reported data from social systems.

Only 7 articles reported both social and environment data (Table 6). The most commonly co-reported social factor was health/safety (n = 5), which was the core focus of several articles measuring environmental health impacts. For several other articles, the social impacts related to economics for otherwise environmental studies. One article [53] reported across a suite of 7 affected factors.

**Table 6:** List of articles and the affected factors reported for environmental and social systems for articles mentioning both environmental and social impacts

Citation and country	Factor	System
Kabir and Bilgi [54]	Atmosphere > Air quality	Environmental
	Health/wellbeing > Health/safety	Social
Moiseenko et al. [55]	Health/Wellbeing > Health/safety	Social
	Surface water > Quality	Environmental
Moiseenko et al. [56]	Health/wellbeing > Health/safety	Social
	Surface water > Quality	Environmental
	Fauna > Individual species	Environmental
	Ecosystems > Quality	Environmental
Rybakov [57]	Health/wellbeing > Health/safety	Social
	Atmosphere > Air quality	Environmental
Saariniemi [53]	Landscapes > Scenic resources	Social
	Economic > Traditional livelihoods	Social
	Services and infrastructure > Infrastructure	Social
	Health/wellbeing > Other	Social
	Ecosystems > Quality	Environmental
	Health/wellbeing > Recreation	Social
	Services and infrastructure > Consumables/subsistence	Social
Semenova [58]	Soil surface > Quality	Environmental
	Health/wellbeing > Health/safety	Social
Wolff and Thomas [59]	Sediment > Quality	Environmental
	Ecosystems > Quality	Environmental
	Economic > Traditional livelihoods	Social

### The measured outcomes/systems

**Fig. 13:**
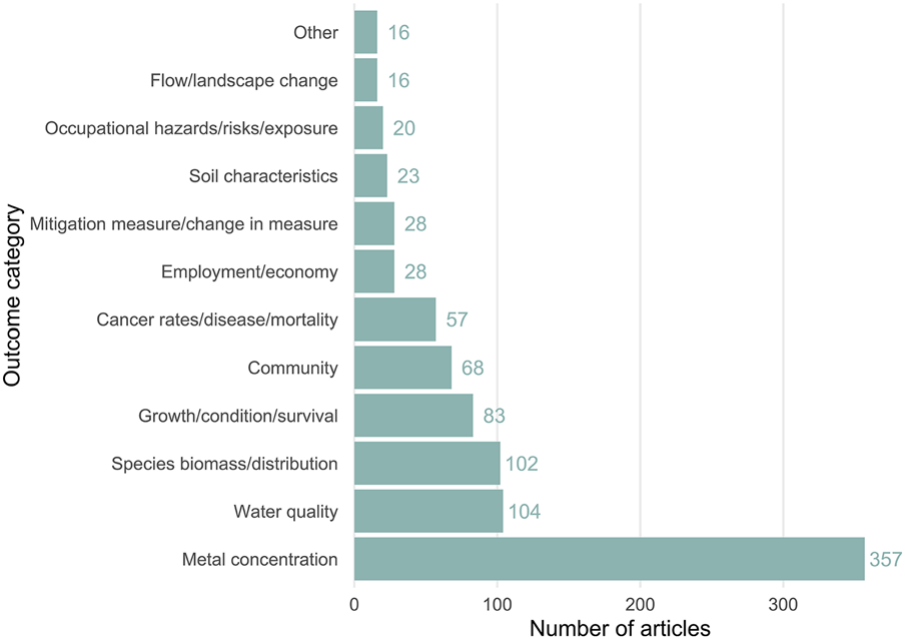
The number of articles reporting measured outcome categories. Note that some articles reported multiple outcomes

Across all articles, the most commonly reported outcome category was metal concentration (n = 357), followed by water quality (n = 104) and species biomass or distribution (n = 102) (see Fig. 13). The most commonly reported social outcome was ‘community’ (i.e. community level social outcome measures) (n = 68), followed by ‘cancer rates/disease/mortality’ (n = 57). The least frequently reported outcome was hydrological flow or landscape change (n = 16).

### Affected system/component/factor

**Fig. 14:**
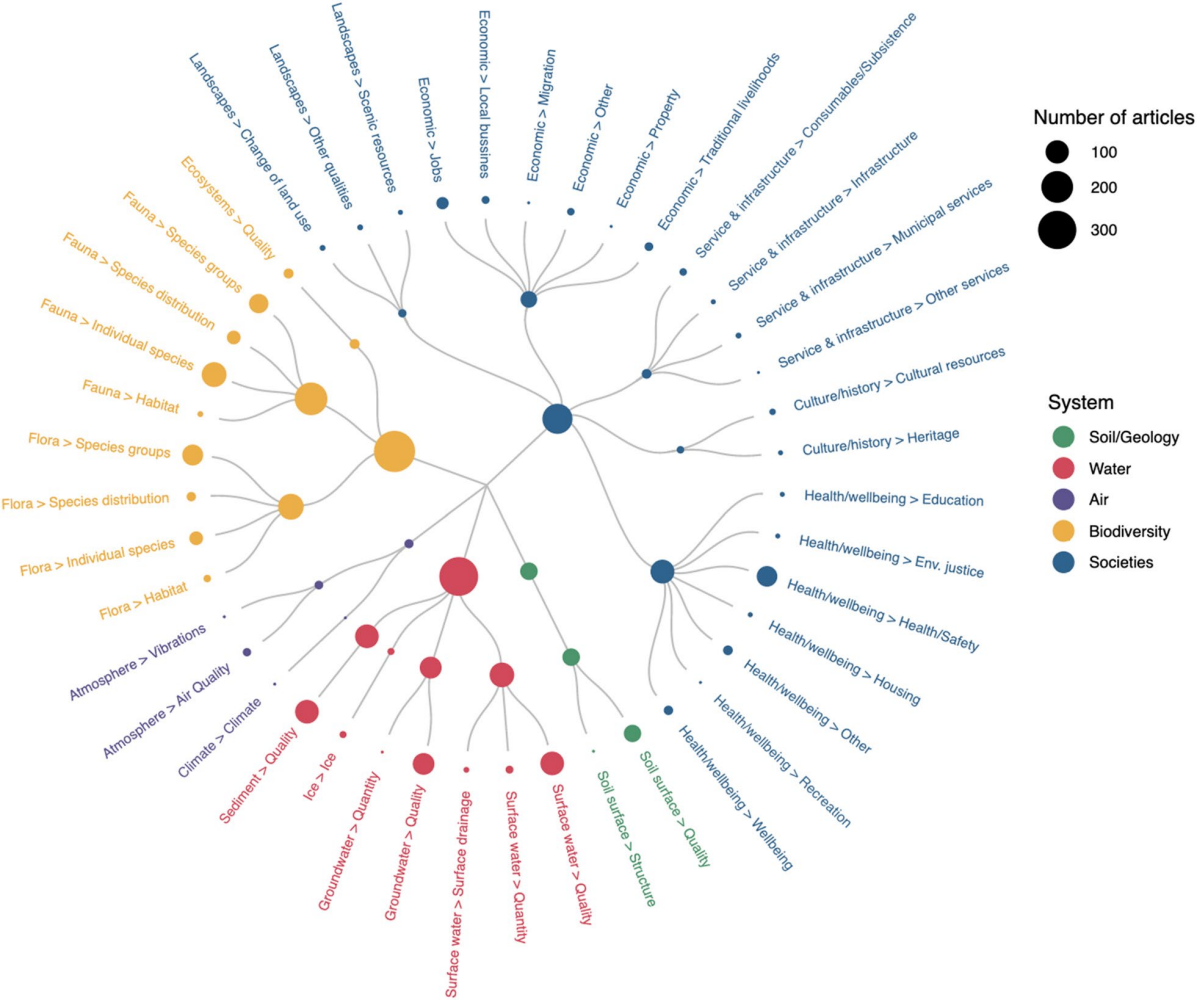
Radial bubble plot of the systems, components and factors affected across the included studies. Systems are depicted by the bubble colour. Bubble size indicates the number of articles. An interactive version is available at the project website; https://3mkproject.github.io/research.html

The systems, components, and factors affected by the mines across included articles is visualised in Fig. 14 and available as an interactive plot on the project website (https://3mkproject.github.io/research.html). This shows that the most commonly reported system was biodiversity (n = 352), followed by water (n = 310), societies (n = 178), and soil/geology (n = 52), with air the least common (n = 10).

Within biodiversity, the most common component was fauna (n = 214), followed by flora (n = 125) and ecosystems (n = 13). Within water, surface, sediment and groundwater were approximately equal (n = 114, 104, and 87 respectively), with ice rather infrequent (n = 5). For societies, health and wellbeing was most common (n = 106), followed by economic (n = 46), service and infrastructure (n = 12), landscapes (n = 8) and culture and history (n = 6). Soil surface was the only component reported for soil/geology articles (n = 52). Within air, atmosphere was most common (n = 9) and climate reported rarely (n = 1).

The most commonly reported factors were individual fauna species (n = 116), water sediment quality (n = 104) and surface water quality (n = 104).

### Country-vs-stage

**Fig. 15:**
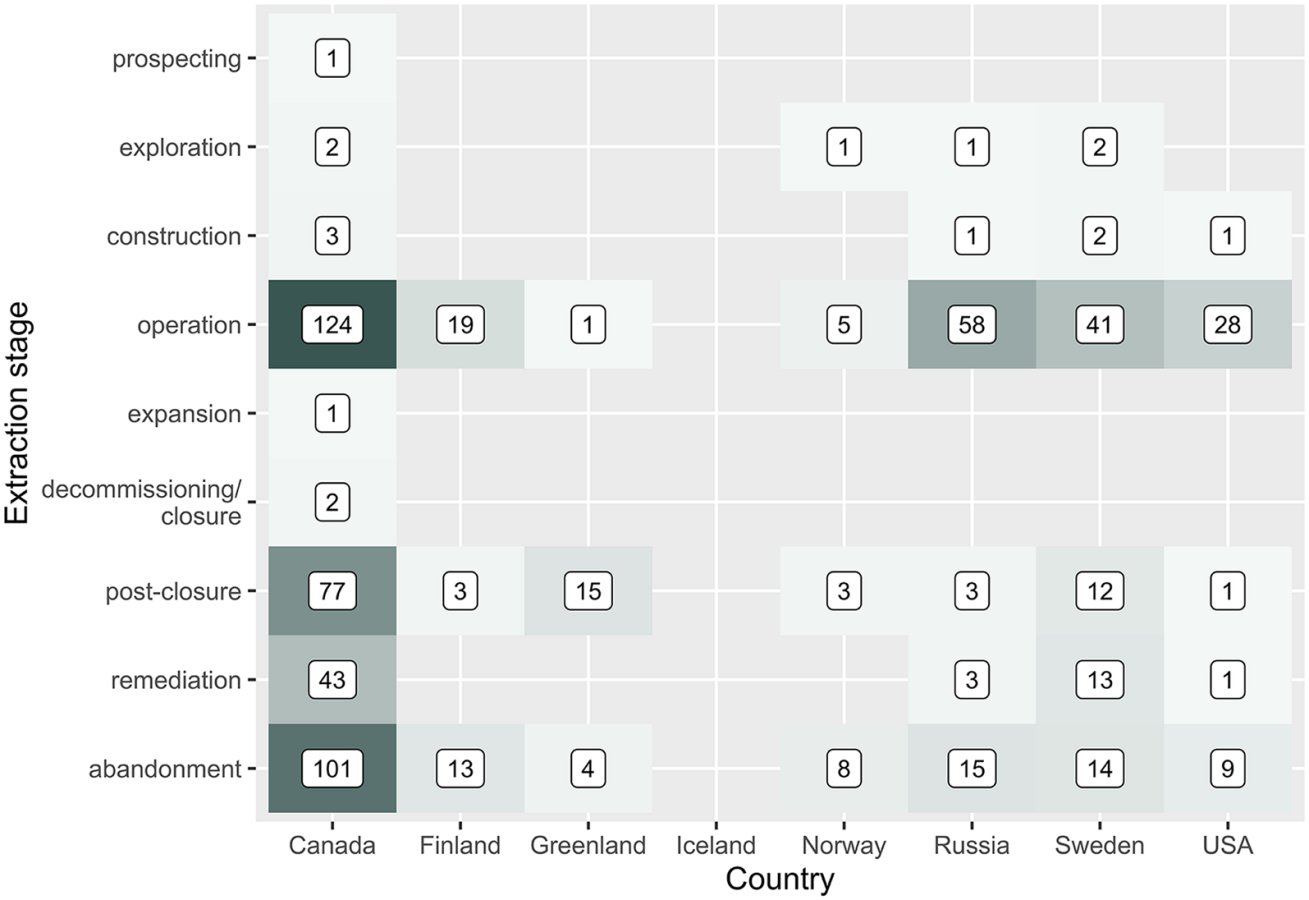
Heat map showing the number of articles across countries and extraction stages

Figure 15 shows how the evidence in the map is distributed across countries and extraction stages, demonstrating that the majority of evidence focuses on operation of mines in Canada. Operation was the most commonly reported stage in Canada, Finland, Russia, Sweden and the USA. In Greenland and Norway post-closure was most frequent. In Russia, the majority of articles focused on operation, with very few on post-closure or remediation relative to Canada and Sweden.

### Limitations of the map

Although our systematic mapping spanned 8 countries across the Arctic and boreal regions, we were restricted in the resources available for screening non-English articles from bibliographic databases (although grey literature searching and screening was performed across multiple languages; Finnish, Swedish, Norwegian, and Danish). A total of 307 full texts could not be screened for this reason (see Additional File 5).

Due to a lack of resources and time during the COVID-19 pandemic, we were unable to perform a search update as planned. As a result, the systematic map represents a snapshot of research from the end of 2018. An updated search of Web of Science Core Collection databases (see Table 2) in June 2021 revealed 2440 records from 2019 to 2021. Assuming the same spread of results across databases, this would have resulted in a total of > 9500 records to screen: almost 25% of the original set of results. Despite this lag from searching to publication, we believe our map is still a valuable assessment of the state of the evidence base, particularly since our assessment of publication rates herein suggested a linear rather than exponential increase in relevant publications over time.

Our screening and data extraction/coding was performed initially using a subset in an attempt to ensure consistency, before proceeding in full with single screening. This is a standard practice amongst systematic reviews and maps published in this journal, and generally considered necessary given the large volumes of evidence common to non-health fields. If we had used full dual screening and data extraction we would have been able to minimise the risk of erroneously excluding some records. However, we believe our methods were both pragmatically necessary and sufficient for the purposes of this mapping exercise.

### Limitations of the evidence base

We identified a large evidence base with a long history. The distribution of studies across outcome measures was relatively even (see Fig. 15), indicating few biases in research attention across the evidence base as a whole. Studies were spread across eligible regions, but a heavy bias in Canada was evident (317/902 data points; 35%). Any further synthesis of these research studies should be sensitive to this bias.

The majority of study designs compared impacted and control samples (396/585 articles, 68%), followed by correlative studies (142/585 articles, 24%), with very few BACI designs (before-after-control-impacts: 6/585 articles, 1%) and no experimental designs. Although this is to be expected, perhaps, given the systems studied, the causal inference of these designs is poor and could be strengthened with randomised control trial study designs—particularly for impact evaluations of mitigation measures which were completely lacking.

Analysis of the validity of studies was not possible in this map, given the wide diversity of data types and study designs (spanning social and environmental science). Internal validity should be assessed in subsequent systematic reviews.

## Conclusion

### Implications for research

Our systematic map database and visualisations of the evidence in heat maps allow readers to gauge gaps and clusters, but here we suggest some areas of the evidence base that are sufficient in number to perhaps be suitable for synthesis in full systematic reviews. This list is based on frequently studied higher level categories (the top 3 affected components and factors). This list is indicative only and should not be taken as a priority list of topics for systematic review.

- 1. On mine operation (n = 428)
  - 1.1. Health/safety (n = 59)
  - 1.2. Air quality (n = 45)
  - 1.3. Fauna individual species (n = 45)
- 2. On post-closure activities (n = 176)
  - 2.1. Fauna individual species (n = 27)
  - 2.2. Surface water quality (n = 22)
  - 2.3. Water sediment quality (n = 20)
- 3. On abandonment (n = 167)
  - 3.1. Fauna individual species (n = 47)
  - 3.2. Surface water quality (n = 40)
  - 3.3. Water sediment quality (n = 39)
- 4. Articles focusing on groundwater quality mitigation measures (n = 60)

Based on our analysis of the evidence base, we also suggest the following topics where there appear to be evidence gaps that warrant further primary research:

1. Studies on earlier stages of resource extraction prior to operation—from prospecting to construction
2. Research on the effectiveness of mitigation measures (including ‘how’ things function)
3. Research employing quasi-experimental (e.g. BACI) and experimental (e.g. randomised control trials) designs for stronger evidence of causality
4. Migration and demography
5. Cumulative impacts and multiple pressures, including effects related to a changing climate
6. Impacts on local and Indigenous communities, especially social, cultural, and health-related

### Implications for policy/management

This systematic map demonstrates that there is a large body of evidence on the impacts of metal mining in Arctic and boreal regions. Many topics within the map constitute potential areas for further synthesis or primary research on the impacts of particular types of mining on certain outcomes (described in *Implications for Research*, above). Relevant funders and decision-makers should ensure they are aware of these gaps and clusters, and commission further research as necessary, making the most of the available evidence. Where large bodies of evidence exist (e.g. operation of mines in Canada), research commissioners should consider whether funding and resources are best placed filling gaps than providing more research on a well-studied topic. Given the inherent connection between mineral development and the Indigenous rightsholders of the north it is also important to ensure that research and synthesis activities are done in ways that involve them and respect their sovereignty, knowledge systems, and cultures.

With efforts to move away from fossil fuels, demand for minerals to support “green” energy technologies is expected to increase. In many parts of the Arctic, these expectations have led to increasing prospecting for minerals and a push for opening new mines. However, mining is often met by protests, especially when new mines or expanded mining activities come into conflict with other land uses and with efforts to protect ecologically valuable nature [60–63]. Furthermore, the process for assessing the impacts of mining have been criticised, with calls for more holistic assessment processes that include meaningful engagement by rightsholders and stakeholders [64] and the whole mining industry is facing increasing distrust [65]. An essential aspect of transparent assessment processes is access to relevant scientific information on all aspects on the implications of mining, and the mapping provided in this paper shows that information on many aspects is limited, especially impacts related to social wellbeing and the interactions between social and environmental factors. Such studies will require approaches that take the whole social-ecological-technological system into account [66], while filling such more holistically-oriented studies with details on specific aspects will require the types of research reviewed in this paper. In addition to identifying critical knowledge gaps, the mapping and potential updates of the map thus serve as an important resource for more holistic environmental and social impact assessment that will be essential for (1) protecting the environment, (2) ensuring that the local social consequences of mining are indeed positive also in the longer term, and (3) helping ensure that the mining permit processes do not stall in drawn-out conflicts due to limited (or contested) knowledge about potential impacts. Better assessment processes will not solve conflict over land use but would at least make decisions about acceptable risks and opportunities more transparent.

Whilst it is expected that most mitigation measures will address the construction and operations phases of a mining project, since this is typically what an Environmental and Social Impact Assessment focuses on, the gaps identified in this systematic map point not only to research gaps but problems with the EIA process itself. For example, EIA legislation is rarely applied to the exploration and development phases of mining, which we found evidence of impacts for. This highlights that there should probably be some legal requirement for analysis and addressing these risks in impact assessments in the future. Policy-makers should be aware that the social impacts from mining in particular begin early on in a project’s lifetime. There is a clear need for more research, and more mitigation, to address these issues.

It is also interesting we found no research examining the EIA process itself, including its effectiveness, and no explicit examination of the proposed mitigation measures from EIAs. Monitoring and evaluation of impact assessments are large gaps in mining legislation and research, and this should be addressed with a clear demand for impact assessment evaluation from decision-makers.

## Supplementary Information

**Additional file 1: **Roses Form

**Additional file 2: **Relevant reviews used for bibliographic checking

**Additional file 3: **Benchmark articles used for search comprehensiveness checking

**Additional file 4: **Benchmark checking results

**Additional file 5: **List of records excluded at full text screening with reasons

**Additional file 6: **List of articles not obtainable at full text

**Additional file 7: **Meta-data extraction and coding form

**Additional file 8: **Systematic map database

**Additional file 9: **Interactive evidence atlas

**Additional file 10: **R script for producing the visualisations

**Additional file 11: **Database of unique mines

## Data Availability

All data are available as supplementary information and via the Open Science Framework project site for 3MK: https://osf.io/cvh3u.
